# MasterOfPores: A Workflow for the Analysis of Oxford Nanopore Direct RNA Sequencing Datasets

**DOI:** 10.3389/fgene.2020.00211

**Published:** 2020-03-17

**Authors:** Luca Cozzuto, Huanle Liu, Leszek P. Pryszcz, Toni Hermoso Pulido, Anna Delgado-Tejedor, Julia Ponomarenko, Eva Maria Novoa

**Affiliations:** ^1^Centre for Genomic Regulation, The Barcelona Institute of Science and Technology, Barcelona, Spain; ^2^International Institute of Molecular and Cell Biology, Warsaw, Poland; ^3^Universitat Pompeu Fabra, Barcelona, Spain; ^4^Department of Neuroscience, Garvan Institute of Medical Research, Darlinghurst, NSW, Australia; ^5^St Vincent’s Clinical School, UNSW Sydney, Darlinghurst, NSW, Australia

**Keywords:** Nextflow, direct RNA sequencing, nanopore, Docker, singularity

## Abstract

The direct RNA sequencing platform offered by Oxford Nanopore Technologies allows for direct measurement of RNA molecules without the need of conversion to complementary DNA, fragmentation or amplification. As such, it is virtually capable of detecting any given RNA modification present in the molecule that is being sequenced, as well as provide polyA tail length estimations at the level of individual RNA molecules. Although this technology has been publicly available since 2017, the complexity of the raw Nanopore data, together with the lack of systematic and reproducible pipelines, have greatly hindered the access of this technology to the general user. Here we address this problem by providing a fully benchmarked workflow for the analysis of direct RNA sequencing reads, termed *MasterOfPores*. The pipeline starts with a pre-processing module, which converts raw current intensities into multiple types of processed data including FASTQ and BAM, providing metrics of the quality of the run, quality-filtering, demultiplexing, base-calling and mapping. In a second step, the pipeline performs downstream analyses of the mapped reads, including prediction of RNA modifications and estimation of polyA tail lengths. Four direct RNA MinION sequencing runs can be fully processed and analyzed in 10 h on 100 CPUs. The pipeline can also be executed in GPU locally or in the cloud, decreasing the run time fourfold. The software is written using the NextFlow framework for parallelization and portability, and relies on Linux containers such as Docker and Singularity for achieving better reproducibility. The *MasterOfPores* workflow can be executed on any Unix-compatible OS on a computer, cluster or cloud without the need of installing any additional software or dependencies, and is freely available in Github (https://github.com/biocorecrg/master_of_pores). This workflow simplifies direct RNA sequencing data analyses, facilitating the study of the (epi)transcriptome at single molecule resolution.

## Introduction

Next generation sequencing (NGS) technologies have revolutionized our understanding of the cell and its biology. However, NGS technologies are heavily limited by their inability to sequence long reads, thus requiring complex bioinformatic algorithms to assemble back the DNA pieces into a full genome or transcriptome. Moreover, NGS technologies require a PCR amplification step, and as such, they are typically blind to DNA or RNA modifications (Novoa et al., 2017).

The field of epitranscriptomics, which studies the biological role of RNA modifications, has experienced an exponential growth in the last few years. Systematic efforts coupling antibody immunoprecipitation or chemical treatment with next-generation sequencing (NGS) have revealed that RNA modifications are much more widespread than originally thought, are reversible (Jia et al., 2011), and can play major regulatory roles in determining cellular fate (Batista et al., 2014), differentiation (Lin et al., 2017; Furlan et al., 2019; Lee et al., 2019) and sex determination (Haussmann et al., 2016; Lence et al., 2016; Kan et al., 2017), among others. However, the lack of selective antibodies and/or chemical treatments that are specific for a given modification have largely hindered our understanding of this pivotal regulatory layer, limiting our ability to produce genome-wide maps for 95% of the currently known RNA modifications (Jonkhout et al., 2017; Boccaletto et al., 2018).

Third-generation sequencing (TGS) platforms, such as the one offered by Oxford Nanopore Technologies (ONT), allow for direct measurement of both DNA and RNA molecules without prior fragmentation or amplification (Brown and Clarke, 2016), thus putting no limit on the length of DNA or RNA molecule that can be sequenced. In the past few years, ONT technology has revolutionized the fields of genomics and (epi)transcriptomics, by showing its wide range of applications in genome assembly (Jain et al., 2018), study of structural variations within genomes (Cretu Stancu et al., 2017), 3′ poly(A) tail length estimation (Krause et al., 2019; Workman et al., 2019), accurate transcriptome profiling (Bolisetty et al., 2015; Sessegolo et al., 2019), identification of novel isoforms (Byrne et al., 2017; Križanovic et al., 2018) and direct identification of DNA and RNA modifications (Carlsen et al., 2014; Simpson et al., 2017; Garalde et al., 2018; Leger et al., 2019; Liu et al., 2019; Parker et al., 2020). Thus, not only this technology overcomes many of the limitations of short-read sequencing, but importantly, it also can directly measure RNA and DNA modifications in their native molecules. Although ONT can potentially address many problems that NGS technologies cannot, the lack of proper standardized pipelines for the analysis of ONT output has greatly limited its reach to the scientific community.

To overcome these limitations, workflow management systems together with Linux containers offer an efficient solution to analyze large-scale datasets in a highly reproducible, scalable and parallelizable manner. In the last year, several workflows to analyze nanopore data have become available, which are aimed at facilitating genome assembly (e.g., Katuali),^1^ genome annotation (e.g., Pinfish^2^) and single nucleotide polymorphism analyses (e.g., NanoPipe^3^). However, none of the current available pipelines cannot be used for the analysis of direct RNA sequencing datasets.

**FIGURE 1:**
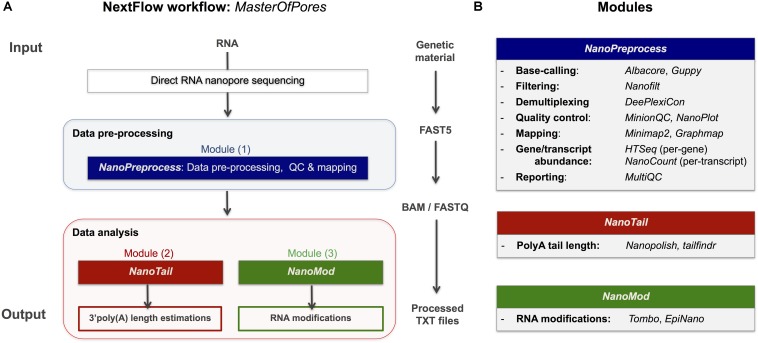
Overview of the *MasterOfPores* workflow for the processing of direct RNA nanopore sequencing datasets. **(A)** Overview of the 4 modules included in the *MasterOfPores* workflow. The pre-processing module (*NanoPreprocess*) accepts both single FAST5 and multi-FAST5 reads and includes 8 main steps: (i) base-calling, (ii) demultiplexing (iii) filtering, (iv) quality control, (v) mapping and (vi) gene or transcript quantification and (vii) final report building. The outputs generated by *NanoPreprocess* (BAM, FastQ and base-called Fast5) are used as input by the subsequent *MasterOfPores* data analysis modules, to predict RNA modifications (*NanoMod*) and polyA tail length estimations (*NanoTail*). **(B)** Detailed description of the individual steps and software used for each of the 4 modules included in *MasterOfPores*.

Here we provide a scalable and parallelizable workflow for the analysis of direct RNA (dRNA) sequencing datasets, termed *MasterOfPores*,^4^ which uses as input raw direct RNA sequencing FAST5 reads, which is a flexible HDF5 format used by ONT to store raw sequencing data, which includes current intensity values, metadata of the sequencing run and base-called fasta sequences, among other features. The *MasterOfPores* workflow performs both data pre-processing (base-calling, quality control, demultiplexing, filtering, mapping, estimation of per-gene or per-transcript abundances) and data analysis (prediction of RNA modifications and estimation of polyA tail lengths) (Figure 1). Thus, the *MasterOfPores* workflow facilitates the analysis of nanopore (epi)transcriptomics sequencing data.

For each step, the workflow extracts metrics which are compiled in a final HTML report that can be easily visualized an analyzed by non-expert bioinformaticians. For each sequencing run, the pipeline produces as output a FASTQ file containing the base-called reads, a BAM file containing the mapped reads, and up to three plain text files containing gene or isoform quantifications, polyA tail length estimations and RNA modification predictions. A direct RNA sequencing run produced by MinION or GridION devices, which typically comprises 1-2M reads, takes ∼2 h to process on a CPU cluster using 100 nodes, and ∼1 h or less on a single GPU (see Table 1 for detailed metrics). Moreover, the pipeline can also be run on the cloud (see section “Running on AWS”).

**TABLE 1:** Comparison of computing time and RAM used to run the pipeline for the four *S. cerevisiae* polyA(+) direct RNA sequencing datasets used in this study.

		Yeast WT *rep1*	Yeast ime△ KO *rep1*	Yeast WT *rep2*	Yeast ime△ KO *rep2*
				
Raw data	Number of reads	1,197,462	694,907	629,270	573,404
**Module (1): NanoPreprocess**					
CPU*	Total time	2 h 13 min	2 h 6 min	2 h 11 min	2 h 1 min
	Total time per 1000 reads (s)	7 s	10 s	12 s	12 s
GPU**	Total time	6 h 44 min	4 h 05 min	3 h 59 min	3 h 19 min
	Total time per 1000 reads (s)	20 s	21 s	23 s	21 s
GPU***	Total time	1 h 8 m	37 min	36 min	30 min
	Total time per 1000 reads (s)	3 s	2 s	2 s	1 s
**Module (2): NanoTail**					
CPU*	Total time	3 h 26 min			
	Total time per 1000 reads (s)	4 s			
**Module (3): NanoMod**					
CPU*	Total time	5 h 40 min			
	Total time per 1000 reads (s)	7 s			

*CPU time computed using a maximum of 100 nodes with 8 CPU per node; **GPU time computed using 1 card GIGABYTE GeForce RTX 1660 Ti; ***GPU time computing using 1 card INNO3D GeForce RTX 2080.

*MasterOfPores* simplifies the analysis of direct RNA sequencing data by providing a containerized pipeline implemented in the NextFlow framework. It is important to note that this approach avoids the heavy-lifting of installing dependencies by the user, and thus, is simple and accessible to any researcher with little bioinformatics expertise. We expect that our workflow will greatly facilitate the access of Nanopore direct RNA sequencing to the community.

## Results

### Overview of the *MasterOfPores* Workflow

Workflow management systems together with Linux containers offer a solution to efficiently analyze large scale datasets in a highly reproducible, scalable and parallelizable manner. During the last decade, an increasing interest in the field has led to the development of different programs such as Snakemake (Köster and Rahmann, 2012), NextFlow (Di Tommaso et al., 2017), Galaxy (Afgan et al., 2018), SciPipe (Lampa et al., 2019) or GenPipes (Bourgey et al., 2019), among others. These tools enable the prototyping and deployment of pipelines by abstracting computational processes and representing pipelines as directed graphs, in which nodes represent tasks to be executed and edges represent either data flow or execution dependencies between different tasks.

Here we chose the workflow framework NextFlow (Di Tommaso et al., 2017) because of its native support of different batch schedulers (SGE, LSF, SLURM, PBS, and HTCondor), cloud platforms (Kubernetes, Amazon AWS, and Google Cloud) and GPU computing, which is crucial for processing huge volumes of data produced by nanopore sequencers. NextFlow has tight integration with lightweight Linux containers, such as Docker and Singularity. Automatic organization of intermediate results produced during the NextFlow pipeline execution allows reducing the complexity of intermediary file names and the possibility of name clashing. Continuous check-pointing with the possibility of resuming failed executions, interoperability and meticulous monitoring and reporting of resource usage are among other thought-after features of NextFlow. The executables of the presented pipeline have been bundled within Docker images accessible at DockerHub that can be converted on the fly into a Singularity image, thus allowing the HPC usage.

The *MasterOfPores* workflow includes all steps needed to process raw FAST5 files produced by Nanopore direct RNA sequencing and executes the following steps, allowing users a choice among different algorithms (Figure 1). The pipeline consists of 3 modules:

(i) *NanoPreprocess*: this module takes as input the raw Fast5 reads and produces as output base-called sequences both in FAST5 and FASTQ formats, as well as alignments in BAM format. The pre-processing module performs base-calling, demultiplexing, filtering, quality control, mapping and gene and/or transcript quantification, generating a final report of the performance and results of each of the steps performed.
(ii) *NanoTail*: this module takes as input the output from the NanoPreprocess module and produces polyA tail length estimations using two different algorithms.
(iii) *NanoMod*: this module takes as input the files generated during the pre-processing step, and produces flat text files with the predicted RNA modifications using two different algorithms.

### Pre-processing Module: *NanoPreprocess*

**FIGURE 2:**
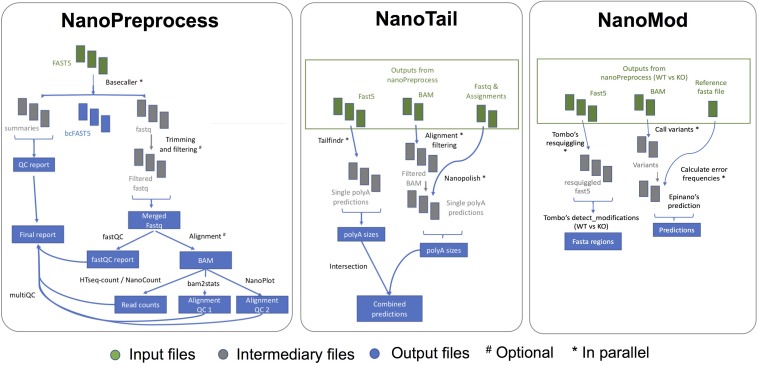
Scheme of the individual steps performed, inputs and outputs of the three modules (NanoPreprocess, NanoTail, and NanoMod) included in *MasterOfPores* workflow. The inputs required by each module are depicted in green, whereas final outputs generated by each module are shown in blue.

**FIGURE 3:**
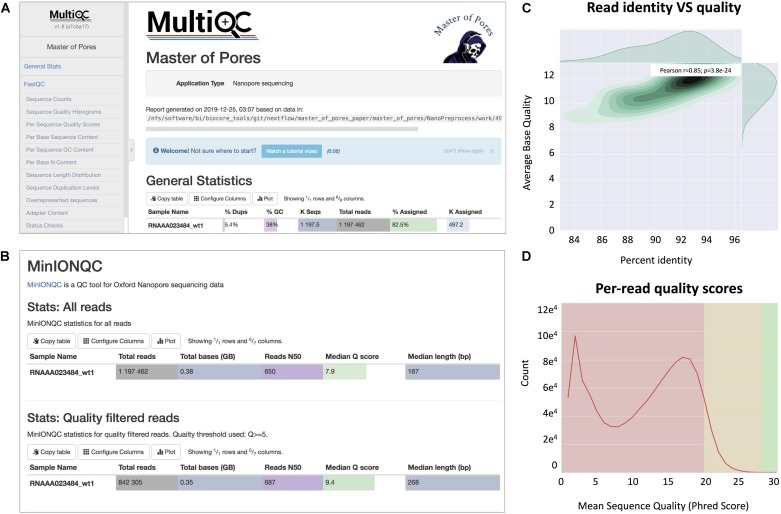
Snapshots of the final report generated by *MasterOfPores*. **(A)** Main menu and overview of the final report generated by *MasterOfPores*. **(B)** The report includes detailed metrics on the input reads (“MinIONQC”), as well as on the mapped reads (“AlignmentQC”). **(C,D)** Example of plots that are included as part of the *MasterOfPores* final report, some of which are generated by integrating Nanoplot **(C)** and FastQC **(D)** software.

The *NanoPreprocess* module consists of 8 main steps (Figure 2):

(i) Read base-calling with the algorithm of choice, using *Albacore*^[Fn footnote5]^ or *Guppy*.^[Fn footnote5]^ This step can be run in parallel and the user can decide the number of files to be processed in a single job by using the command *–granularity*.
(ii) Demultiplexing of the reads using *DeePlexiCon* (Smith et al., 2019). This step is optional, and can only be used if the libraries have been barcoded using the oligonucleotides used to train the deep neural classifier^6^
(iii) Filtering of the resulting fastq files using *Nanofilt* (De Coster et al., 2018). This step is optional and can be run in parallel.
(iv) Quality control of the base-called data, using *MinIONQC* (Lanfear et al., 2019) and FastQC.^7^
(v) Read mapping to the reference genome or transcriptome, using *minimap2*^8^ or *graphmap2*.^9^
(vi) Quality control on the alignment, using *NanoPlot*^10^ and *bam2stat*s.^11^
(vii) Gene or transcript quantification, using *HTSeq (Anders et al., 2015)* or *NanoCount*.^12^ The latter estimates transcript abundance using an expectation-maximization algorithm. *NanoCount* will be run if reads have been mapped to the transcriptome, using the flag *–reference_type* transcriptome, whereas *HTSeq* will be employed to quantify per-gene counts if the reads have been mapped to the genome.
(viii) Final report of the data processing using *multiQC*^13^ that combines the single quality controls done previously, as well as global run statistics (Figure 3).

### Data Analysis Modules: *NanoTail* and *NanoMod*

The *MasterOfPores* pipeline contains two additional modules for the downstream analyses of the mapped reads, namely *NanoTail* and *NanoMod*, which provide polyA tail length estimations and RNA modification predictions, respectively (Figure 2). The modules can be run using as input the output from the *NanoPreprocess* module.

The *NanoTail* module estimates polyA tail lengths using *Nanopolish*^14^ and *TailfindR*,^15^ producing a plain text file with polyA tail length estimations for each read, computed using both algorithms. The correlation between the two algorithms is also reported as a plot.

The *NanoMod* module predicts RNA modifications using *Tombo*^16^ and *EpiNano*,^17^ producing a plain text files with the predicted sites by each algorithm. The NanoMod module is run “paired mode,” i.e., providing two conditions, as both *EpiNano* and *Tombo* identify RNA modifications by comparing two conditions.

### Running *MasterOfPores:* Installation, Input, Parameters and Output

To run *MasterOfPores*, the following steps are required:

(i) Install NextFlow (version 19.10.0):
  $ *curl -s https://get.nextflow.io | bash*

(ii) Clone the MasterOfPores repository:
  $ *git clone –depth 1 https://github.com/biocorecrg/master_of_pores.git*

(iii) Install Docker or Singularity (for Singularity, version 2.6.1 and Docker 19.03 or later are required):
  Docker: https://docs.docker.com/install/
    Singularity: https://sylabs.io/guides/2.6/user-guide/quick_start.html#quick-installation-steps

(iv) Download Nanopore base-calling algorithms: *guppy* with or without GPU support and or the albacore Wheel file (a standard built-package format used for Python distributions) and install them inside the *bin* folder inside the MasterOfPores directory. The users can place their preferred version of guppy and/or albacore in the *bin* folder (in the example below, albacore version 2.1.7 and guppy 3.1.5).
  $ *cd master_of_pores/NanoPreprocess/bin*
    $ *tar -zvxf ont-guppy_3.1.5_linux64.tar.gz*
    $ *ln -s ont-guppy_3.1.5_linux64/ont-guppy/bin/guppy_^∗^.*
    $ *pip3 install –target* = *./albacore ont_albacore-2.1.7-cp36-cp36m-manylinux1_x86_64.whl*
    $ *ln -s albacore/bin/multi_to_single_fast5*
    $ *ln -s albacore/bin/read_fast5_basecaller.py*

(v) Optional step: install CUDA drivers (only needed for GPU support):
  https://docs.nvidia.com/cuda/cuda-installation-guide-linux/index.html

(vi) Run the pre-processing step of the pipeline (using singularity *or* docker):
  $ *cd./*
    $ nextflow run nanopreprocess.nf-with-singularity
    or
    $ *nextflow run nanopreprocess.nf-with-docker*

(vii) Run polyA tail estimation module
  $ *cd./NanoTail*
    $ *nextflow run nanotail.nf-bg-with-singularity –input_folders “.NanoPreprocess/output/RNA^∗^”*

(viii) Run RNA modification prediction module
  $ cd./*NanoMod*
    $ nextflow run nanomod.nf *-with-singularityinput_path “.NanoPreprocess/output/”*

The *NanoPreprocess* module can handle both single- and multi-FAST5 reads as input. To execute the workflow, several parameters can be defined by the user, including the choice of the basecaller (albacore or guppy), mapper (minimap2 or graphmap2), as well as their command line options. If these are not specified by the user, the workflow will be run with default parameter settings specified in the params.config file (Table 2). The final report includes four different types of metrics: (i) *General statistics* of the input, including the total number of reads, GC content and number of identical base-called sequences; (ii) *Per-read statistics* of the input data, including scatterplots of the average read length versus sequence identity, the histogram of read lengths, and the correlation between read quality and identity; (iii) *Alignment statistics*, including the total number of mapped reads, the total number of mapped bases, the average length of mapped reads, and the mean sequence identity; (iv) *Quality filtering statistics*, including the number of filtered reads, median Q-score and read length, compared to those observed in all sequenced reads*;* and (v) *Per-read analysis of biases*, including information on duplicated reads, over-represented reads and possible adapter sequences (Figure 3). The final outputs of this module include:

**TABLE 2:** Settings and parameters that can be customized to run the NanoPreprocess module of the MasterOfPores workflow.

	Parameter	Description of the parameter	Default Values
RUN_INFO	kit	Sequencing kit used (SQK-RNA001 or SQK-RNA002)	SQK-RNA002
	flowcell	flowcell type	FLO-MIN106
	fast5	fast5 files including the path	“$baseDir/data/multifast/^∗^.fast5”
	annotation	annotation file (GTF) including path	“”
	reference	reference genome or transcriptome sequence	“$baseDir/anno/curlcake_constructs.fasta.gz”
	ref_type	reference type (genome or transcriptome)	“genome”
RUN_SETUP	seqtype	sequence type (RNA or DNA)	“RNA”
	output	Output folder	“$baseDir/output”
	qualityqc	Quality threshold for QC	5
	granularity	Number of files analyzed per process	“”
DEMULTIPLEXING	demultiplexer demultiplex_opt	Option to run demultiplexing, in case the run is barcoded (ON or OFF) choose between different pre-trained models	“OFF” “-m pAmps-final-actrun_newdata_nanopore_UResNet20v2_ model.030.h5”
BASE-CALLING	basecaller	Can be: albacore/guppy	“guppy”
	basecaller_opt	Command line options for basecalling	“”
	GPU	Whether or not using GPU (ON or OFF)	“OFF”
FILTERING	filter filter_opt	Can be empty, OFF or nanofilt command line options for filtering	“” “”
MAPPING	mapper	Can be minimap2 or graphmap2 or empty	minimap2
	mapper_opt	Command line options for mapping	“-uf -k14”
	map_type reference_type	Can spliced or unspliced can be transcriptome, genome or both	“spliced” “genome”
GENE COUNTING	counter	Option to compute per-gene or per-transcript counts from the mapped BAM file (YES or NO)	“YES”
	counter_opt	Command line options for counting. Of note, per-gene counts will be computed using HTSeq if reference_type is “genome,” or computed using NanoCount if reference_type is “transcriptome”	“”
REPORTING	email	Email (to receive the report when finished)	“”

– Basecalled fast5 files within the “fast5_files” folder.
– Filtered fastq files within “fastq_files” folder.
– QC reports within “QC” folder.
– Final report within “report” folder.
– Aligned reads in sorted BAM files within the “aln” folder.
– Read counts within the “counts” folder.

The *NanoMod* module requires two samples to detect RNA modifications, typically wild-type and knock-out (or knock-down) matched conditions. The user must provide a tab-delimited file (–comparison “comparison.tsv”) indicating which input file is the wild-type condition and which one is the knock-out or knock-down condition (see, for example^18^), which is specified in the parameter file. The *NanoMod* module will output the results into two different folders:

– RNA modification results predicted using *Tombo* in the “Tombo” folder
– RNA modification results predicted using *EpiNano* in the “EpiNano” folder

The *NanoTail* module will output the results into three different folders:

– PolyA tail length estimates predicted using *Nanopolish*, in the “Nanopolish” folder.
– PolyA tail length estimates predicted using *tailfindR*, in the “Tailfindr” folder.
– In this module, an additional “NanoMod_final” folder is generated, containing combined *Nanopolish* and *tailfindR* estimates of polyA tail lengths, as well as information regarding the geneID or transcriptID where the read is mapped to.

### Running *MasterOfPores* on the Cloud (AWS Batch and AWS EC2)

Nanopore sequencing allows for real-time sequencing of samples. While GridION devices come with built-in GPUs that allows live base-calling, smaller MinION devices do not have built-in CPU or GPU. Thus, the user has to connect the MinION to a computer with sufficient CPU/GPU capabilities, or run base-calling after the sequencing. In all these contexts, the possibility of running the *MasterOfPores* pipeline on the cloud presents a useful alternative.

The Amazon Web Services (AWS) Batch is a computing service that enables users to submit jobs to a cloud-based user-defined infrastructure, which can be easily set up via either code-based definitions or a web-based interface. Computation nodes can be allocated in advance or according to resource availability. Cloud infrastructure can be also deployed or dismantled on demand using automation tools, such as CloudFormation or Terraform.

Here we show that the MasterOfPores pipeline can be successfully implemented on the cloud, and provide the Terraform script for running *MasterOfPores* on the AWS Batch CPU environments, available in the GitHub repository.^19^ To run the pipeline using the AWS Batch, the users needs to change only a few parameters related to their accounts in a configuration file. The pipeline can be run from either a local workstation or an Amazon EC2 entrypoint instance initiated for this purpose (we recommend the latter). Data to be analyzed can be uploaded to an Amazon S3 storage bucket.

Similarly, we also tested whether our pipeline could be run in Amazon Web Services (AWS) Elastic Compute Cloud (EC2), which is one of the most popular cloud services (Supplementary Table S1). Compared to AWS Batch, to run any workflow in AWS EC2, the user must first create an Amazon Machine Image (AMI). The AMI can be created using the same instructions as provided in Supplementary File S1, starting from the official Ubuntu 18.04 LTS AMI, and including both Docker and Singularity software with NVIDIA libraries support. Here we show that the resulting image can be used to run the *MasterOfPores* workflow with NVIDIA Tesla V100 GPU cards. Automation scripts to run *MasterOfPores* in AWS EC2 can be found in the GitHub repository.^20^

### Test Case: Analysis of *Saccharomyces cerevisiae* SK1 PolyA(+) RNA

#### Running the *MasterOfPores* Pipeline on *S. cerevisiae* PolyA(+) RNA

To benchmark the performance of the *MasterOfPores* workflow, we employed four publicly available direct RNA sequencing runs of polyA(+)-selected *S. cerevisiae* WT and ime4△ strains, in biological replicates, which had been sequenced using MinION and GridION devices, producing a total of ∼3 million reads (Table 1). We used up to 100 nodes with 8 CPUs for testing the base-calling in CPU mode and 1 node with 1 GPU card for testing the base-calling in GPU mode (Table 1).

The MasterOfPores *NanoPreprocess* module was ran using guppy version 3.1.5 as the base-caller and minimap2 version 2.17 as the mapping algorithm. Reads were filtered by running nanofilt with the options “-q 0 –headcrop 5 –tailcrop 3 –readtype 1D”. Filtered reads were mapped to the yeast SK1 fasta genome. Specifically, the command that was executed to run the pipeline with these settings was:

*$ cd master_of_pores/NanoPreprocess* *$ nextflow run nanopreprocess.nf –basecaller guppy –seqtype RNA \* *–fast5 “FOLDERNAME/^∗^.fast5” –demultiplexing “OFF” \* *–map_type “spliced” –mapper_opt “-uf -k14” \* *–reference genome.fa.gz –mapper minimap2 –ref_type “genome”\* *–filter nanofilt –filter_opt “-q 0 –headcrop 5 –tailcrop 3 –readtype 1D*”.

Then, the two data analysis modules were executed as follows:

*$ nextflow run nanotail.nf –input_folders “./NanoPreprocess/output/^∗^” \* *–nanopolish_opt “” –tailfindr_opt “” –reference “genome.fa.gz”*

*$ nextflow run nanomod.nf –input_path “./NanoPreprocess/output/” \* *–comparison “./comparison.tsv” –reference “genome.fa.gz” \* *–tombo_opt “–num-bases 5” –epinano_opt “”*

#### Benchmarking the Time Used for the Analysis of *S. cerevisiae* PolyA(+) RNA

Here we have tested the pipeline using both CPU and GPU computing. Specifically, we ran the pipeline on the following configurations: (i) a single CPU node (e.g., emulating the computing time on a single laptop); (ii) a CPU cluster with 100 nodes; (iii) a single mid-range GPU card (RTX2080); and (iv) a single high-end GPU card (GTX1080 Ti). We found that the computing time required to run the pipeline on a single GPU card was significantly lower than the running time in parallel on a high performance CPU cluster with 100 nodes, 8 cores per node (Table 1, see also Supplementary Table S1). Moreover, we found that the computing time of the NanoPreprocess module can be significantly reduced depending on the GPU card (base-calling step was ∼2X faster for GTX1080 Ti than for RTX2080).

#### Reporting Resources Used for the Analysis of *S. cerevisiae* PolyA(+) RNA

**FIGURE 4:**
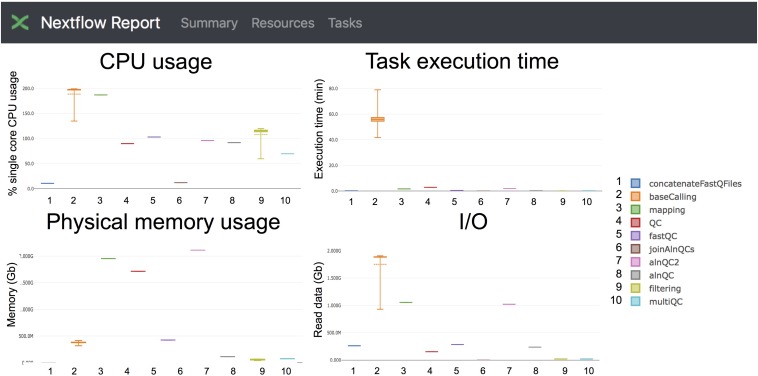
Snapshot of the NextFlow resources report. The report includes detailed information of the computing resources and time needed to execute each of the modules of the pipeline. Base-calling and mapping are the most CPU demanding tasks. The base-calling step is the longest to run, whereas mapping and generation of alignment QC metrics are the most memory-demanding tasks.

Taking advantage of the NextFlow reporting functions, the pipeline can produce detailed reports on the time and resources consumed by each process (Figure 4), in addition to the output files (bam, fastq) and final report (html), if the workflow is executed with parameters *-with-report* (formatted report) or*-with-trace* (plain text report). Running the base-calling on each multi-fast5 file in parallel on our dataset showed that the most memory intensive tasks (about 5 Gbytes) were the mapping step (using minimap2) and the quality control step (using *Nanoplot*) (Table 3), while the most CPU-intensive and time-consuming step (∼80 min) was the base-calling (using *Guppy*) (Table 4).

**TABLE 3:** RAM peak (Mbytes) used by each of the pre-processing module.

Sample	Number of reads (M)	Base-calling	Mapping	QC	FastQC	alnQC	alnQC2	Filtering	Counting	MultiQC
**wt1**	1.2	578	4,517	2,751	283	109	4,891	76	34	76
**wt2**	0.6	458	2,129	1,651	520	39	4,751	69	34	57
**ko1**	0.7	417	1,954	1,715	427	115	2,111	70	34	77
**ko2**	0.6	480	1,771	1,400	494	49	2,266	69	34	75

**TABLE 4:** CPU time peak (min) used by each of the steps of the pre-processing module.

Sample	Number of reads (M)	Base- calling	Mapping	QC	FastQC	alnQC	alnQC2	Filtering	Counting	MultiQC
**wt1**	1.2	33	1	4	1	1	2	1	9	1
**wt2**	0.6	67	1	3	1	1	1	1	4	1
**ko1**	0.7	79	2	3	1	1	2	1	6	1
**ko2**	0.6	66	1	3	1	1	1	1	4	1

Finally, we should note that the latest (19.10.0) version of NextFlow allows the user to control the execution of a pipeline remotely. To enable this feature, the user needs to login to the https://tower.nf/website developed by the NextFlow authors and retrieve a token for communicating with the pipeline. For doing that, the user should set this token as an environmental variable and run the pipeline as follows:

*$ export TOWER_ACCESS_TOKEN* = *YOUR_TOKEN* *$ cd master_of_pores/NanoPreprocess* *$ nextflow run nanopreprocess.nf -with-docker -with-report -bg -with-tower*

## Discussion

The direct RNA sequencing technology developed by Oxford Nanopore technologies (ONT) offers the possibility of sequencing native RNA molecules, allowing to investigate the (epi)transcriptome at an unprecedented resolution, in full-length RNA molecules and in its native context. Although the direct RNA sequencing library preparation kit was made available in April 2017, only a modest number of researchers have started to adopt this new technology, partly due to the complexity of analyzing the resulting raw FAST5 data. Moreover, even in those cases when specific software and tools have been made available, the users typically experience many difficulties in installing dependencies and running the software. To overcome these issues and facilitate the data analysis of direct RNA sequencing to the general user, we propose the use of NextFlow workflows.

Specifically, we propose the use of *MasterOfPores* workflow for the analysis of direct RNA sequencing datasets, which is a containerized pipeline implemented in the NextFlow framework. *MasterOfPores* can handle both single- and multi-FAST5 reads as input, is highly customizable by the user (Table 2) and produces informative detailed reports on both the FAST5 data processing and analysis (MultiQC report, Figure 3) as well as on the computing resources used to perform each step (NextFlow report, see Figure 4). Thus, the current outputs of the *MasterOfPores* workflow include: (i) base-called FAST5 files, (ii) base-called fastq file, (iii) sorted BAM file containing mapped reads, (iv) per-gene or per-transcript counts (depending on algorithm choice), (v) MultiQC report, (vi) NextFlow report, (vii) per-read polyA tail length estimations, including the correlation of predictions using two distinct algorithms, and (viii) per-site RNA modification predictions, including a final plain text file containing the consensus sites predicted by two distinct algorithms.

The process of Nanopore read base-calling, that is, converting ion current changes into the sequence of RNA/DNA bases, has significantly improved during the last few years, mainly due to the adoption of deep learning approaches, such as the use of convolutional neural networks (CNNs) and recurrent neural networks (RNNs), which are currently the most commonly used strategies for base-calling. The adoption of RNN and CNN-based base-calling algorithms has led to a dramatic improvement in base-calling accuracy. However, this has come at the expense of a higher computational cost: only 5–10 reads can be base-called on 1 CPU core per second using the latest versions of the base-calling algorithms. The use of graphic processing units (GPUs) can greatly accelerate certain CPU-intensive computational tasks, thus allowing to process 50–500 reads per second (Supplementary Table S1). We therefore developed our pipeline for both CPU and GPU computing. Moreover, we provide the GPU-enabled docker image and detailed information on how to setup the GPU computing (see section: “Running MasterOfPores”). We encourage users to adopt the GPU computing for the analysis of Nanopore sequencing data whenever possible, as this option is both more time- and cost-efficient.

## Materials and Methods

### Code Availability

The pipeline is publicly available at https://github.com/biocorecrg/master_of_pores under an MIT license. The example input data as well as expected outputs are included in the GitHub repository. Detailed information on program versions used can be found in the GitHub repository. EpiNano was modified from its original version (1.0) to decrease the computing time of the pipeline (EpiNano version 1.1, available at https://github.com/enovoa/EpiNano).

### Documentation Availability

Detailed documentation on how to install and use the pipeline can be found at: https://biocorecrg.github.io/master_of_pores/

### Availability of Docker Files and Docker Images

The pipeline uses software that is embedded within Docker containers. Docker files are available in the GitHub repository.^21^ The pipeline retrieves a specific Docker image from DockerHub. In particular, the workflow retrieves four distinct images: one for basecalling,^22^ one for demultiplexing,^23^ one for pre-processing^24^ and one for measuring polyA tail lengths and detecting RNA modifications.^25^

### Integration of Base-Calling Algorithms in the Docker Images

Due to the terms and conditions that users agree to when purchasing Nanopore products, we are not allowed to distribute Nanopore software (binaries or in packaged form like docker images). While the original version of the *MasterOfPores* pipeline includes both guppy and albacore, we are not legally allowed to distribute it with the binaries. Therefore, here we only make available a version where the binaries must be downloaded and placed into a specific folder by the user. We expect future versions of *MasterOfPores* will include these programs within the docker image once this issue is solved.

### CPU and GPU Computing Time and Resources

The *MasterOfPores* workflow was tested both locally (using either CPU or GPU) as well as in the cloud (AWS). Computing times for each mode are shown in Table 1. CPU time was determined using a maximum of 100 nodes simultaneously with maximum 8 cores CPU per node (2.8–3.5 GHz, 80–130 Watt). GPU time was computed using either GIGABYTE GeForce RTX 1660 Ti (1536 CUDA cores @ 1770 MHz with 6GB of GDDR6 vRAM memory, 120 Watt) or INNO3D GeForce RTX 2080 (2944 CUDA cores @ 1710 MHz with 8 GB of GDDR6 vRAM memory, 225 Watt) or NVIDIA Tesla V100 (5120 CUDA cores + 640 Tensor cores @ 1462 MHz with 16 GB of HBM2 memory). For GPU computing, both system memory (RAM) and GPU memory (vRAM) are used. Base-calling with guppy typically uses 1 or 4.2 Gb of vRAM in fast and high accuracy mode, respectively. As a result, only one base-calling process can be performed on above mentioned cards in high accuracy mode at given time. The execution time in the AWS EC2 p3.2xlarge instance involves reading files already placed in a previously set-up S3 storage bucket but not writing back output results into it.

## Data Availability Statement

Direct RNA sequencing datasets for *Saccharomyces cerevisiae* SK1 PolyA(+) RNA were taken from publicly available GEO datasets (GSE126213).

## Author Contributions

LC wrote the pipeline. HL optimized EpiNano code to incorporate it into the pipeline and tested the pipeline. LP tested the pipeline and implemented GPU computing for containers. TP implemented and tested the workflow for AWS cloud computing. AD-T tested the pipeline with MinION, GridION, and PromethION sequencing runs and helped with the optimization of the NanoPreprocess module. JP and EN supervised the work. LC and EN made figures and tables. EN conceived the project. LC, JP, and EN wrote the manuscript, with contributions from all authors.

## Conflict of Interest

EN has received travel and accommodation expenses to speak at Oxford Nanopore Technologies conferences. The remaining authors declare that the research was conducted in the absence of any commercial or financial relationships that could be construed as a potential conflict of interest.

## Footnotes

1 https://github.com/nanoporetech/katuali

2 https://github.com/nanoporetech/pipeline-pinfish-analysis

3 https://github.com/IOB-Muenster/nanopipe2

4 https://biocorecrg.github.io/master_of_pores/

5 https://nanoporetech.com

6 https://github.com/Psy-Fer/deeplexicon

7 http://www.bioinformatics.babraham.ac.uk/projects/fastqc

8 https://github.com/lh3/minimap2

9 https://github.com/lbcb-sci/graphmap2

10 https://github.com/wdecoster/NanoPlot

11 https://github.com/lpryszcz/bin

12 https://github.com/a-slide/NanoCount

13 https://github.com/ewels/MultiQC

14 https://github.com/jts/nanopolish

15 https://github.com/adnaniazi/tailfindr

16 https://github.com/nanoporetech/tombo

17 https://github.com/enovoa/EpiNano

18 https://github.com/biocorecrg/master_of_pores/NanoMod/comparison.tsv

19 https://biocorecrg.github.io/master_of_pores/

20 https://biocorecrg.github.io/master_of_pores/

21 https://github.com/biocorecrg/master_of_pores/tree/master/docker/

22 https://cloud.docker.com/u/biocorecrg/repository/docker/biocorecrg/mopbasecall

23 https://hub.docker.com/repository/docker/biocorecrg/mopdem

24 https://cloud.docker.com/u/biocorecrg/repository/docker/biocorecrg/mopprepr

25 https://cloud.docker.com/u/biocorecrg/repository/docker/biocorecrg/mopmod
